# The Filtering of the Posturographic Signals Shows the Age Related Features

**DOI:** 10.1155/2014/403893

**Published:** 2014-12-17

**Authors:** Krzysztof Piotr Michalak, Anna Przekoracka-Krawczyk, Paweł Nawrot, Piotr Woźniak, Peter Vieregge

**Affiliations:** ^1^Laboratory of Vision Science and Optometry, Faculty of Physics, Adam Mickiewicz University of Poznań, Umultowska Street 85, 61-614 Poznań, Poland; ^2^Polish Mother's Memorial Hospital-Research Institute, Rzgowska Street 281/289, Łódź, Poland; ^3^Neurology Clinic, Klinikum Lippe, Rintelner Street 85, 32657 Lemgo, Germany

## Abstract

*Objective*. Lower frequencies of slow oscillations of the posturographic signals can be removed using high-pass filtering. This procedure releases postural reflexes possessing higher frequencies and lower amplitude range. Mutual dependence between the *x* and *y* components of posturographic signals was analyzed using principal component analysis (PCA). The posturographic signals of old patients with idiopathic gait disturbance were compared with the control group of similar age and with younger patients. There was also the analysis of the influence of the eyes state (open versus closed) and the head position (normal or bent back). The statistically significant differences in the mutual dependence between *x* and *y* components between the groups of patients were analyzed using MANOVA. The significant differences were observed mainly in the range of filter frequencies *f* = 0.1–1.5 Hz and *f* = 2.2–5.5 Hz with a maximum effect at approximately 4-5 Hz. A detailed post-hoc analysis is also presented. The differences in the higher frequency range suggest the main disturbance to be connected with the spinal reflexes. Visual and vestibular support appear insufficient for postural stability control in the idiopathic gait disturbance group. The results suggest that idiopathic gait disturbance is the final stage of the aging process of postural system.

## 1. Introduction

All the postural reflexes responsible for standing in the upright position are represented in posturographic signal which reflects the process of moving the center of pressure (COP) to the platform in the quiet stance [1]. The posturographic signal consists of 2 main parts: (a) a slow drift of the center of mass that is represented by low frequencies in Fourier's spectrum (*f*<  ~0.6 Hz) [2] and (b) small corrections in higher frequencies that reflect postural reflexes. Postural reflexes can be decomposed into two parts: spinal reflexes and central nervous system corrections [3]. The regulatory processes from the spinal reflexes are faster since they are progressing through a smaller number of synapses and action potentials have a shorter way to travel. Central regulation takes place especially in the extrapyramidal tracts and in the cerebellum. They create the main part responsible for transferring visual, vestibular, and proprioceptive information. Central regulation is slightly slower because of the higher complexity of all the processes that are involved in the information transfer. Two components can be distinguished in CNS: the visual and the vestibular one. When one of these components deteriorates its working, the function of the other becomes more important [4–7].

The postural control is affected especially by two factors: (a) the speed at which action potentials are transmitted from and to the muscles and (b) the quality of the transferred information. The transmission speed becomes slower with age. The speed depends on the quality of myelin sheaths surrounding the proprioceptive and motor fibers and on the velocity of the contraction response of the muscular fibers in response to incoming stimuli.

In turn, the following factors influence the quality of information: (a) the precision of the stretching information from the muscle receptors, (b) the number of motoneurons, sensory cells, and intermediate neurons in the given part of the nervous system, (c) the precision of information being transferred through the intermediate neurons, and (d) the energetic processes in neural fibers restoring the resting potential of the nerve cell. Dysfunctions in any of these elements can influence the final postural stability control. Static posturography analysis is, however, still unable to distinguish precisely between the functioning of all these factors. Thus, one of the purposes of this paper is looking for the possibility of performing such analyses using advanced calculation techniques.

## 2. The Posturographic Signal

The posturographic signal represents all the regulatory processes that keep the body in the upright position. Disturbances in different regulatory areas can be, however, expected to exhibit different attributes in the posturographic signal. For example, disturbances in spinal reflexes are expected to be evident in higher frequency range due to the shorter duration of spinal corrections. Oppositely, the dysfunctions of the central nervous system are expected to be visible in the lower frequencies due to longer response times. The muscle fiber contraction being the answer for a single stimulus has approximately the shape of a reversed cosine. It begins about 20–30 ms after the stimulus reaches the muscle fiber. The maximum contraction takes place after about 100 ms and it vanishes after about 250 ms. The action potential is transmitted in motoneurons and proprioceptive neurons at a speed of 50–60 m/s. Depending on the distance of travel, it corresponds to the total transmission time of about 10–40 ms.

Based on the presented information, it can be concluded that the total time of postural correction responses is about 160–1000 ms. It corresponds approximately to a frequency of 1–6 Hz. Moreover, it can also be concluded that the spinal reflexes that use a smaller number of synapses can be observed in the range of 2–6 Hz. The effects of the central regulation are approximated to occur in the range of about 1–3 Hz due to greater number of synapses and longer transmission time.

The center of mass of the human body is located at a height of 0.8-0.9 m. The frequency of vibrations of the inverted pendulum can be estimated to be about 0.6-0.7 Hz [2]. Thus, oscillations ranging from 0.6 to 1 Hz are related to substantial fluctuations of the upright position of the human body, implying serious anomalies of the postural control. Oppositely, oscillations <0.6 Hz are related to a slow center of mass (COM) drift rather than to postural reflexes. When compared to fluctuations at higher frequency range, these fluctuations are often of higher amplitude and because of it, they mask proper reflexes of the posture control. Posturographic signals are characterized in their Fourier spectra by the decreasing amplitudes while frequencies increase. Thus, analysis of posturographic signals requires, first of all, removing the lower frequencies in order to uncover higher frequency corrections with lower amplitudes.

### 2.1. The Division of Posturographic Signals into Their Components

The attempt to separate posturographic signals into the separate components relating individual regulatory processes of the CNS is a very complex task. Amoud et al. [8] divided the posturographic signals of old and young participants, using empirical mode decomposition (EMD), into 4 components. The mean frequencies of the successive components were 0.5 Hz, 1–1.5 Hz, 3-4 Hz, and 6–8 Hz. The age related differences were observed in the parameters “area of Hilbert transform” and “average rotation frequency.” A similar decomposition was also presented by Ramdani et al. [9]. Amoud et al. [8] showed the differences between age groups both using basic-EMD with separate analysis for *x* and *y* components and using complex-EMD where the interdependence between *x* and *y* components is not removed. Discrete wavelet decomposition and modified PCA to decompose the stabilogram into three components were proposed by Maatar et al. [10]. He called these components: trend, rambling, and trembling.

Loram et al. [11–13] presented another type of analysis of posturographic signal. He showed using an advanced EMG method that the calf muscles are actively adjusted 2.6 times per second and about 2.8 times per unidirectional sway of the center of mass (COM). These alternating, small movements provide impulsive ballistic regulation of COM. This experiment shows that the activity of proper posturographic reflexes should be found in the Fourier spectrum in the range of *f* > 2 Hz and the modifications of the unidirectional large-amplitude sway are very small when compared to the size of this sway.

The use of the digital filters to the posturographic signal is very sparse. Horlings et al. [6] showed that persons with the bilateral proprioceptive loss of the lower legs are characterized by other pelvises to shoulder characteristics in the high-pass filter posturographic signals of *f* > 3 Hz.

In the present study, a number of digital high-pass filters ranging from *f* = 0.05 to 6 Hz were analyzed. Next, principal component analysis (PCA) was used to analyze the common relation between *x* and *y* components and to partially remove the noise. PCA is a method that depends only partially on the spectrum of given components. The task that appears in this method concerns the building of the matrix being orthogonally rotated using PCA. Possessing the standard posturographic signal, it is possible to include the components *x* and *y* into this matrix (spatial decomposition) or to include the same components shifted in time (temporal decomposition). Up to now, PCA method was purely used in the analysis of posturographic signals. The PCA method has been used to compare independence of different parameters estimated from nonfiltered signals, rather than to apply the spatiotemporal decomposition of the signal itself [14–16].

The removal of slow frequencies that reflect a slow center of mass drift was not performed in earlier studies. Therefore, the filtering procedure and other methods of the signal decomposition are the promising tools in developing new, innovative diagnosis methods.

## 3. Materials and Methods

### 3.1. Subjects

Posturographic signals were recorded in the Clinics of Neurology in the Medical University of Luebeck. The investigation was performed according to the principles of the Declaration of Helsinki and was approved by Ethics Committee at the University of Luebeck. The informed consent was obtained from subjects after the aim of the procedure was explained.

384 participants were included in the study. The patients were divided into 4 groups: 3 age-fitted groups of normal, healthy patients (groups B1–B3) and a group of older patients with idiopathic gait disturbance (group A). Group A included 54 patients of average age 81.9 ± 6.5. Next, group B1 included 98 normal patients of average age 76.5 ± 4.1, 193 patients of average age 61.6 ± 5.2 joined group B2, and finally 39 normal, healthy patients of average age 30.1 ± 5.6 were included in group B3. The patients in group A either (a) reported nonspecific gait disturbances resulting in stumbling or falling in the period of the previous six months or (b) were neurologically diagnosed as having an unexplained and unclassified gait disorder. Patients were rejected if the gait disorder could be explained by one or more of the following reasons: paraparesis, hemiparesis, tetraspasticy, or tetraparesis, any kind of myelopathic, cerebellar, myopathic, vestibular, brainstem, or neuropathic lesions, any degenerative disease of the peripheral or central motor system, intake of CNS-relevant drugs, and any medical, dermatologic, or orthopedic dysfunction interfering with gait.

Normal, healthy patients who did not exhibit any deviations in the neurological examination joined groups B1–B3. The healthy persons had to present an inconspicuous gait pattern and had to be able to perform six tandem steps without deviation during at least one of two subsequent trials.

### 3.2. Apparatus and Procedure

The participants stood upright, with their feet 80 mm apart on a force-measuring platform. The center of pressure displacement was recorded under static conditions in the anteroposterior (*y* coordinate) and mediolateral (*x* coordinate) directions. Signal recording procedure was performed 4 times for each participant under different conditions in respect to eyes state and head position: (1) eyes open (E_O_) and normal position of the head (head normal H_N_); (2) eyes closed (E_C_) and H_N_; (3) E_O_ and standing with head maximally pulled back (head bent back H_BB_; vestibular stimuli are partially excluded from the body balance); (4) E_C_ and H_BB_. In the E_O_ condition, the participants fixed their gaze on a small dot drawn on the wall at gaze level and at the distance of *L* = 2 m. Pressure forces were digitized using the sampling rate of *f* = 50 Hz. The data were recorded for 30 s, but only the last 20.48 s were taken into further analysis. The initial fragment of 9.52 s was treated as a posture stabilization period [17] and rejected. Each sweep had, therefore, 1024 2-dimensional data points.

### 3.3. Signal Analysis

#### 3.3.1. Filtering and Principal Component Analysis

First, the components *x* and *y* of the signal were filtered using high-pass filters which removed the low-frequency components of the signals. Signals were filtered using FIR digital filter (Matlab) with a filter order *R* = 300. Analysis was applied for 30 different high-pass filter frequencies ranging from *f* = 0.05 Hz to 6 Hz. After filtering, the *x* and *y* signals were normalized: $x_{n}=\left(x-\bar{x}\right)/\sigma_{x}$ and $y_{n}=\left(y-\bar{y}\right)/\sigma_{y}$. Next, the PCA method (Principal Component Analysis) was used. PCA is a commonly accepted method of feature extraction and noise reduction [18]. It is recommended by Oliveira et al. [19] as a more suitable method for estimating the common relation between *x* and *y* components of the posturographic signal than classic linear regression.

As a result of PCA method, the orthogonal rotation of the data matrix was performed in order to maximize variations in the successive columns of resultant matrix. In three successive experiments, the matrix analyzed using PCA was constructed from 4, 6, and 8 columns. These three experiments are called *T*_2_, *T*_3_, and *T*_4_. The index *i* at the letter *T*_*i*_ denotes the number of successive samples of the signals *x*_*n*_ and *y*_*n*_ which create the successive columns in the input PCA matrix. The presented approach is the generalization of the approach presented in [20] where the input PCA matrix was built from 4 columns: I: *x*_*n*_(1~1023),  II: *x*_*n*_(2~1024), III: *y*_*n*_(1~1023), and IV: *y*_*n*_(2~1024). Columns I-II and III-IV reflect the same signals being shifted in time by one sample (i.e., *t* = 0.02 s). Because of it, these columns are strongly correlated.

In the case of experiment *T*_*i*_, the input PCA matrix consists of *i* vectors *x*_*n*_ shifted by 1 sample to each other and of *i* vectors *y*_*n*_ shifted by 1 sample to each other: *x*_*n*_(1~1024 − *i* + 1),  *x*_*n*_(2~1024 − *i* + 2),…, *x*_*n*_(*i*, 1024), *y*_*n*_(1~1024 − *i* + 1), *y*_*n*_(2~1024 − *i* + 2),…, *y*_*n*_(*i*, 1024). The *i* successive samples of the given signal *x*_*n*_ or *y*_*n*_ are strongly correlated; however, the correlation decreases with an increasing distance between samples. Applying the PCA method to the presented composition of the PCA matrix gives, as a result, the signal possessing *i*-times reduced noise in the first and second column after orthogonal rotation and mainly the noise in the remaining *i* − 2  columns. Increasing *i* causes, as a result, better noise reduction. However, if *i* is increasing, some part of the signal begins to be included in the third and next columns of the rotated matrix. The noise reduction is connected with higher signal reduction being stored in columns I and II. This reduced part of the signal is included mainly in the column III.

The application of *i* samples corresponds approximately to the application of the signal filtering using the filter frequency *f* = *f*_*s*_/(2*i*), where *f*_*s*_ is the sampling frequency of the signal [21]. In the case of the posturographic signals, the often procedure consists in preliminary low-pass filtering using *f* = 10 Hz that removes mainly the noise. Applying *i* = 2,3, and 4 for *f*_*s*_ = 50 Hz signals corresponds approximately to the low-pass filtering procedure using approximately *f*_*f*_ = 12.5 Hz, 8.33 Hz, and 6.25 Hz.

After applying the PCA method to the 2*i*-column matrix, the resultant, orthogonally rotated matrix includes mainly the *x* and *y* components in columns I and II, while columns III, IV and the others contain mainly the noise. The column III stores some small part of both signals, as well. The singular values (*S*) of the resultant matrix are approximately proportional to the standard deviations of the successive columns. Two parameters were analyzed in detail: the ratio between column II of *S* (*S*_II_) and column I (*S*_I_) (*s*_2_ = *S*_II_/*S*_I_), which refers to the mutual relation between the components *x*_*n*_ and *y*_*n*_ and the ratio between columns III and I (*s*_3_ = *S*_III_/*S*_I_) which refers to the remaining part of the signal and noise. When the parameter *s*_2_ is equal to about 1, then there is no relation between the components *x*_*n*_ and *y*_*n*_. The lower the *s*_2_, the greater the relation between components *x*_*n*_ and *y*_*n*_.

**Figure 1:**
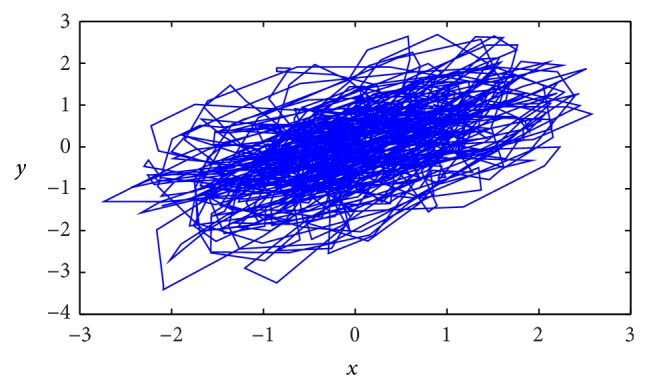
Example of the posturographic signal after 2.6 Hz high-pass filtering. The low frequency and large-amplitude oscillations are removed uncovering the relation between *x*_*n*_ and *y*_*n*_ components. After the PCA rotation, the first column *S*_1_ represents the long diagonal of the path area and the second column (*S*_2_) represents the short diagonal.

Figure 1 shows the example of the high-pass filtered posturographic signal using filter frequency *f* = 2.6 Hz in which high relation between *x*_*n*_ and *y*_*n*_ is observed. *x*_*n*_ and *y*_*n*_ components are visible to be dependent each to other. This figure shows also the necessity of the preliminary normalization of the components. If one of the components *x*_*n*_/*y*_*n*_ would be stronger than the second one, the ratio between *S*_II_ and *S*_I_ in PCA method would refer mainly to the ratio between them and not to their common dependence.

**Figure 2:**
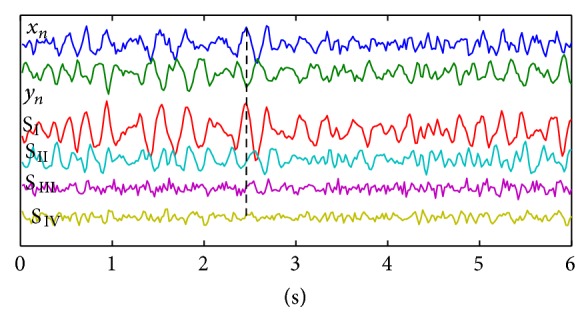
Example of the decomposition of the posturographic signal using spatiotemporal decomposition. The input PCA matrix was built from 4 columns: I: *x*_*n*_(1~1023), II: *x*_*n*_(2~1024), III: *y*_*n*_(1~1023), and IV: *y*_*n*_(2~1024). Columns I-II and III-IV reflect the same signals being shifted in time by one sample (i.e., *t* = 0.02 s). The two upper signals represent the *x*_*n*_ and *y*_*n*_ signals after high-pass filtering *f* = 3 Hz. The 4 lower signals are the PCA columns after rotation. The *S*_I_ and *S*_II_ represent mainly the signal. *S*_III_ and *S*_IV_ represent mainly the noise. The synchronous deflection in *x*_*n*_ and *y*_*n*_ is connected with high amplitude of the deflection in *S*_I_ (see dashed line).

Figure 2 shows the exemplary results of the PCA decomposition. Two upper signals represent the *x*_*n*_ and *y*_*n*_ components before orthogonal rotation and the lower 4 signals present the result of the PCA rotation for *i* = 2. It can be observed that the common oscillation of both *x*_*n*_ and *y*_*n*_ components results in high oscillation in the first PCA component *S*_I_.

The experiment presented in [20] has shown that high-pass filtering was able to remove the low-frequency and high amplitude trend and has uncovered the relation between *x* and *y* components of the posturographic signals in the range of about 2–5 Hz. The current compares the results obtained for different number of successive samples used in the PCA method: *i* = 2,…, 4. Additionally the parameter *s*_3_ is analyzed.

## 4. Results

The analysis shows the parameters *s*_2_ and *s*_3_ which are defined above. MANOVA analysis was performed separately for the set of *s*_2_ and *s*_3_ values. The analysis took into consideration two factors: 4 patient groups (A/B_1–3_) and 4 registering conditions (E_O_H_N_, E_O_H_BB_, E_C_H_N_, and E_C_H_BB_). The analysis was performed separately for every used high-pass filter frequency (30 values ranging from *f* = 0.05 to 6 Hz).

**Figure 3:**
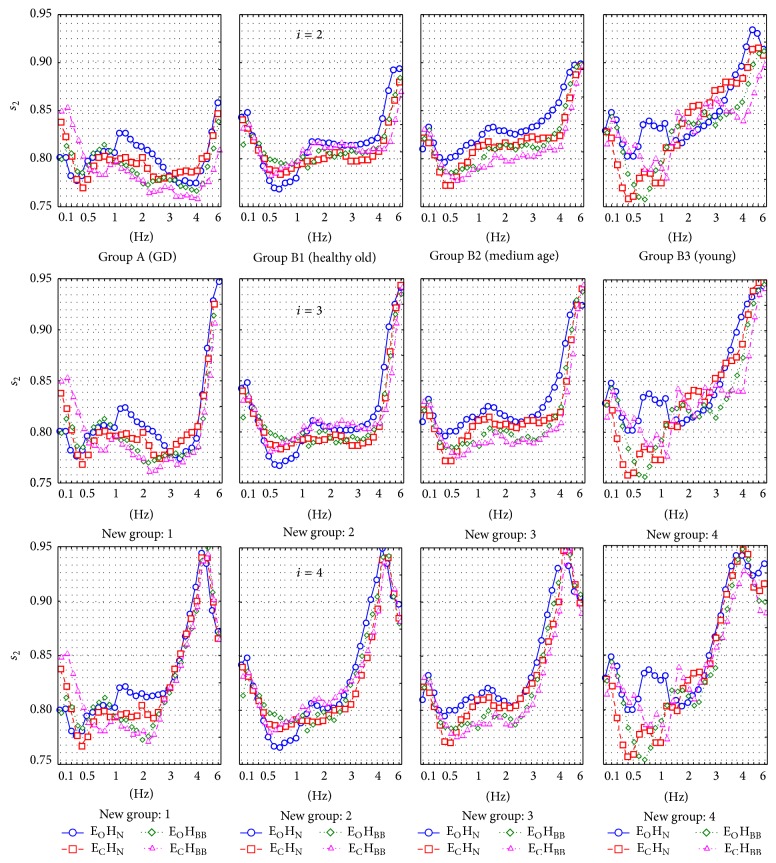
The detailed *s*_2_ values obtained in successive patient groups for different registration methods. Three rows represent different number of columns (*i*) in PCA method.

**Figure 4:**
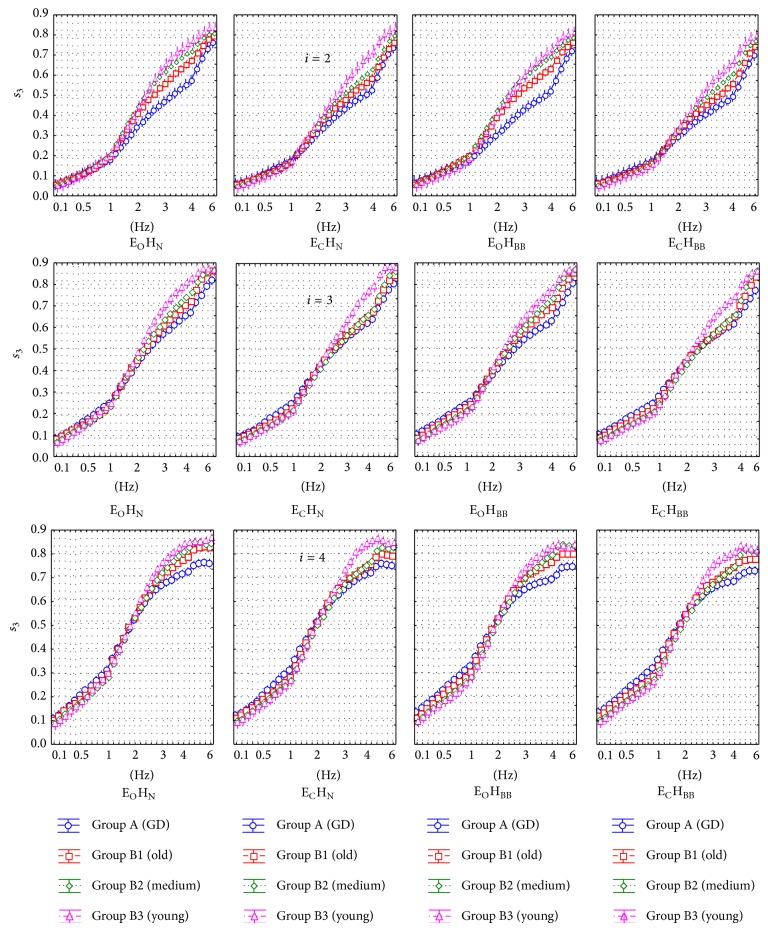
The detailed *s*_3_ values obtained in successive patient groups for different registration methods. Three rows represent different number of columns (*i*) in PCA method.

Figure 3 presents the results for the parameter *s*_2_ and Figure 4 shows the results for the parameter *s*_3_.

Successive subfigures show the results for 3 experiments *T*_2_, *T*_3_, and *T*_4_ in which *i* = 2,3, and 4 were used. Results are presented separately for every filter frequency, eyes/head condition, and age group. These figures show 2 main filter ranges in which differences between groups are especially observed. The first range is about 0.1–1.5 Hz and the second range is about 2–5 Hz.

**Figure 5:**
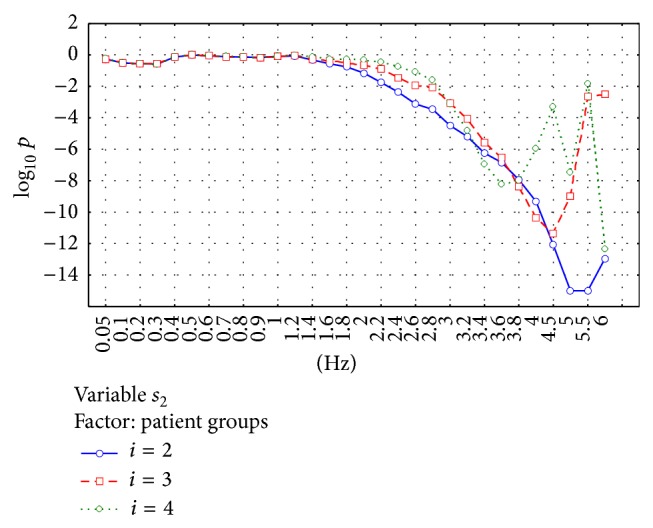
Analysis of the parameter *s*_2_. The figure shows the statistical significance *p* of the main effect in MANOVA analysis for the factor “patient groups.” Logarithmic scale is used. Starting from about 2.4 Hz, an increase in the filter frequency causes the increase in the ability of *s*_2_ to separate different age groups. While building PCA matrix, the use of *i* = 2 gives relative best results.

**Figure 6:**
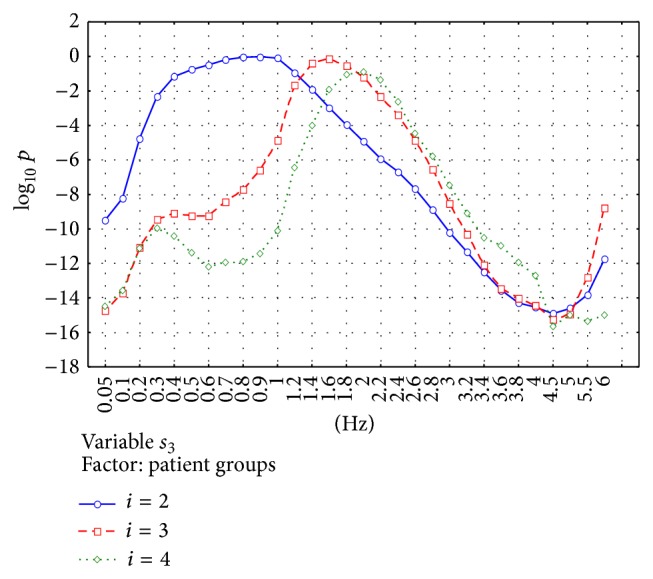
Analysis of the parameter *s*_3_. The figure shows the value *p* of the statistical significance of the main effect in MANOVA analysis for the factor “patient groups.” Logarithmic scale is used. Building PCA using *i* = 2 gives different results compared to using *i* = 3 or 4. In the case of *i* = 3 and 4, the difference between patient groups is strongly significant in the range of 0.1–1.5 Hz which is not observed for *i* = 2. In the range *f* > 2 Hz, the significance is increasing for all *i* = 2, 3, and 4 up to *f* = 4.5 Hz.

**Figure 7:**
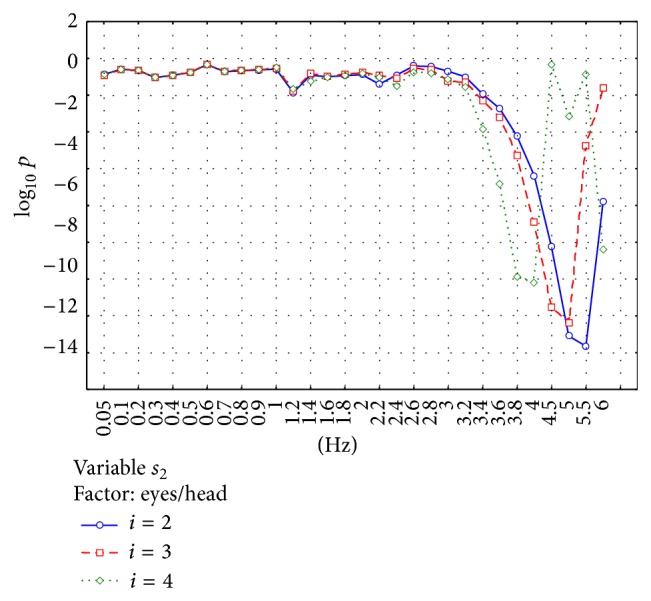
Analysis of the parameter *s*_2_. The figure shows the statistical significance *p* of the main effect in MANOVA analysis for the factor “eyes/head state.” The differences between registration conditions are especially visible for the high-pass filters *f* ≥ 3.4 Hz.

**Figure 8:**
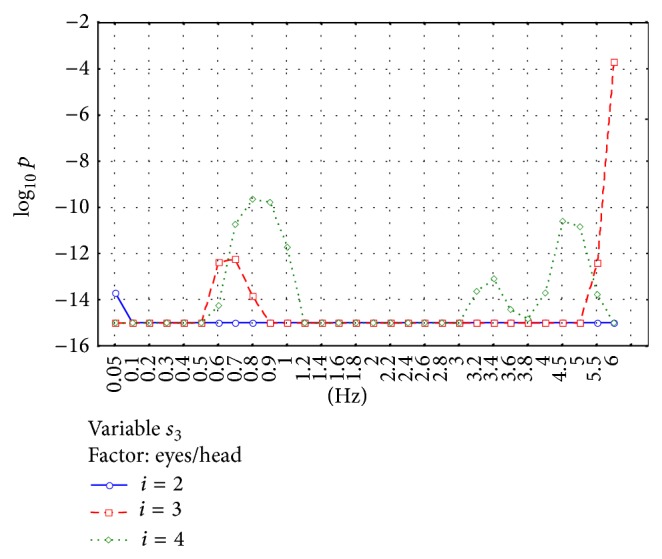
Analysis of the parameter *s*_3_. The figure shows the high statistical significance *p* of the main effect in MANOVA analysis for the factor “eyes/head state.” The very high significance is observed for every filter frequency used. The values for eyes closed (E_C_H_N_ and E_C_H_BB_) are as a rule lower than for open eyes.

The significance of the observed differences was analyzed using ANOVA analysis. Two factors were analyzed: “patient groups” and “eyes/head position.” Figures 5–8 show the significance of differences in the logarithmic scale between successive groups and registration conditions for the variables *s*_2_ and *s*_3_. The lowest value in the OY axis “−15” refers to the significance being lower than *P* < 10^−15^.

Figure 5 shows that the significance of the variable *s*_2_ increases with high-pass filter frequency used. The best significance is observed for the PCA matrix being built using *i* = 2, in general. The significant difference is observed for the filter frequency *f* > 2 Hz and reaches its maximum at *f* = 5 Hz.

Figure 6 shows the same analysis for *s*_3_. The parameter *s*_3_ is characterized, in general, by the better discrimination power between patient groups than *s*_2_. Figure 4 shows the general tendency for *s*_3_ to increase with age in the range *f* = 0.1–1 Hz and to decrease with age in the range *f* = 2–6 Hz. Building PCA using *i* = 2 gives different results compared to using *i* = 3 or 4. In the second case, the difference between patient groups is strongly significant in the range of *f* = 0.1–1.5 Hz which is not observed for *i* = 2. This phenomenon can be explained by lower value of low-pass filter frequency that corresponds to the given value of  *i*. Older persons are characterized by higher mean amplitude of the oscillations in the range 6–20 Hz [20]. When using *i* = 3 or 4, more significant part of the signals *x*_*n*_/*y*_*n*_ is removed from the first and second component and transferred to the third component of the resultant PCA matrix. It causes the increase in *s*_3_ value for the lower filter frequencies.

Figure 7 shows the significance of the *s*_2_ differences between registration methods applied. The results for successive *i* values are similar; the significance is observed for *f* ≥ 3.4 Hz. The tendency for *s*_2_ significance is observed quicker to increase and to reach the maximum when *i* increases from *i* = 2 to 4.

Similar analysis for the variable *s*_3_ presented in Figure 8 shows that the differences between registration methods are strongly significant in all the ranges of the used filter frequencies. The differences are observed especially between eyes open and eyes closed: *s*_3_ is lower for E_C_ condition.

### 4.1. Aging Process in the Post Hoc Analysis

The *s*_2_ results are very similar for *i* = 2,…, 4, in general. The differences occur for filter frequencies *f* > 4 Hz because the low-pass filter frequency corresponding to the given *i* becomes lower (*f*_2_≅12.5 Hz for *i* = 2, *f*_3_≅8.3 Hz for *i* = 3, and *f*_4_≅6.25 Hz for *i* = 4). The post hoc analysis for *s*_2_ concerns the parameter *i* = 2 which exhibits relative most strong significance as presented in Figure 5. The neighboring age groups are especially analyzed.

A detailed comparison between subjects in group B3 (mean age 30 yrs) and group B2 (mean age 61 yrs) shows the highest statistical significance for the registration pattern E_C_H_N_ (*P* < 0.01 for *f* = 4-5 Hz). This result suggests that the role of labyrinth in the postural stability becomes more important when the patient has the eyes closed. The decrease in *s*_2_ if postural control is only supported by the labyrinth suggests that, in the age range from 30 to 60 years, the aging process of the vestibular system deteriorates postural control to a higher degree than the aging process of the visual system. A less important role of the deterioration of visual support may be explained by a lowering of the *s*_2_ parameter in the E_O_H_N_ registration method (*P* < 0.05 for *f* = 4-5 Hz). In turn, *s*_2_ is becoming lower for E_C_H_BB_ (*P* < 0.05 for *f* = 2.6–3.2 Hz). This may suggest that other modulations compared to the visual or vestibular ones may also be weakened (possible in the cerebellum and/or spinal cord). The lower range of frequency, when compared to the range 4-5 Hz, may be the result of higher, thus slower, oscillations while E_C_H_BB_ registering or alternatively may suggest a weakening of some higher levels of regulation.

Comparison between group B1 (mean 76 y) and group B2 (mean 61 y) shows that significant differences occur while performing the classic registration pattern E_O_H_N_ (*P* < 0.05 for *f* = 3.8–5 Hz). It can be concluded that the visual modulation is becoming weakened at this stage of the aging process followed by a weakening of vestibular modulation, but to a lesser extent. It can be observed in group B2 that the visual and vestibular systems working together make it possible to maintain the “juvenile” regulation pattern. This is not observed in B1 group where *s*_2_ is lowered approximately to a similar level for each registration method.

Comparison between groups A (gait dist.) and B1 (healthy 76 y) shows a further lowering of *s*_2_ for all registration methods (E_O_H_N_: *P* < 0.05 for *f* = 3–6 Hz, *P* < 0.01 for 4-5 Hz; E_O_H_BB_: *P* < 0.05 for *f* = 3.8–6 Hz; E_C_H_BB_: *P* < 0.05 for *f* = 2.2–4 Hz). The differences are statistically insignificant for E_C_H_N_ only. The presented results suggest a further general deterioration in the functioning of segmental correction reflexes on postural stability and a further weakening of the influence of the visual system as well as a slightly lesser weakening of the vestibular system.

The results for *s*_3_ are even more significant than for *s*_2_. Thus, the value of *s*_3_ can be a good discriminator of aging process. The interpretation of the results is, however, ambiguous. Based on *s*_3_, it is difficult to interpret the results and to conclude about the functioning of different parts of postural control system. It is dedicated for further research.

## 5. Discussion

The presented results for high-pass filtered posturographic signals let us suggest that acquisition of the filtering for the posturographic signals makes it potentially possible to obtain valuable diagnostic parameters. It can be expected that different parameters calculated for filtered signals will be probably more discriminatory than those for nonfiltered signals.

Though the filtering is unable to make a complete division of the signal into components coming from different parts of the postural system, it is still possible to find statistically significant differences in different patient groups. Filtering is able to remove major slow oscillations possessing high amplitude and to release the effects of the postural reflexes that possess the spectrum in the range 2–6 Hz. In the present experiment the differences were observed mainly for the *f* > 2 Hz frequency.

The filter order is an important parameter in the process of filtering. The maximum possible filter order is 1/3 of the signal length [22]. It was *R*_max⁡_ = 1024/3 = 341 in our analyzed signals. The higher filter order denotes the more sharp border of low-frequency cutoff in the spectrum. A filter order (*R* = 300) was used in the current study in order to make the filtering effective. It must be pointed out, however, that in the case of other acquisition time and/or sampling frequency, the filter order giving the same filtering parameters as in the current paper would be different than *R* = 300.

The presented results make it possible to determine the mean intensity of the aging process of the postural system. Between 30 (B3) and 60 yrs (B2), the deterioration of the vestibular component is more visible than that of the visual one. Hytonen et al. [23] showed that older persons rely more on visual support to keep stable posture. Next, between 60 (B2) and 76 yrs (B1), a further weakening of two analyzed components is observed, with the faster progression in weakening of the visual component. In group B1, the effects of the visual and/or the vestibular system on the *s*_2_ are small. It can be concluded that, at this point, they are not able to improve the strongly deteriorated proprioceptive system.

The integrity of the CNS determines the demand of the attention support to maintain proper posture [24]. Jacobs and Horak [3] claimed that conscious attention plays an important role in postural stability in the elderly, which is manifested by deteriorating posturographic parameters during mental tasks. The present paper considers idiopathic gait disturbances to be the next stage in the deterioration of postural functions. As a result, postural stability becomes more difficult to sustain even when the patient is fully concentrated.

It must be stressed, however, that the results obtained in the present paper should be treated as only pilot ones. *s*_2_ and *s*_3_ are the parameters that only partially describe the postural system. It is probable that other parameters will better discriminate between different groups of patients and registering conditions.

Also, one should remember that it is possible to use more advanced methods which divide posturographic signals into their components and which remove noise. Registration with higher sampling frequencies and lower noise levels will ensure greater precision and accuracy of low-amplitude and high-frequency components in the signal and will allow for expanding the range of filters above the 6 Hz that was used in the present paper.
